# Supplementary figures and images for: On the development of sleep states in the first weeks of life
Source: PLoS One. 2019 Oct 29;14(10):e0224521. doi: 10.1371/journal.pone.0224521 (PMC6818777; doi:10.1371/journal.pone.0224521)

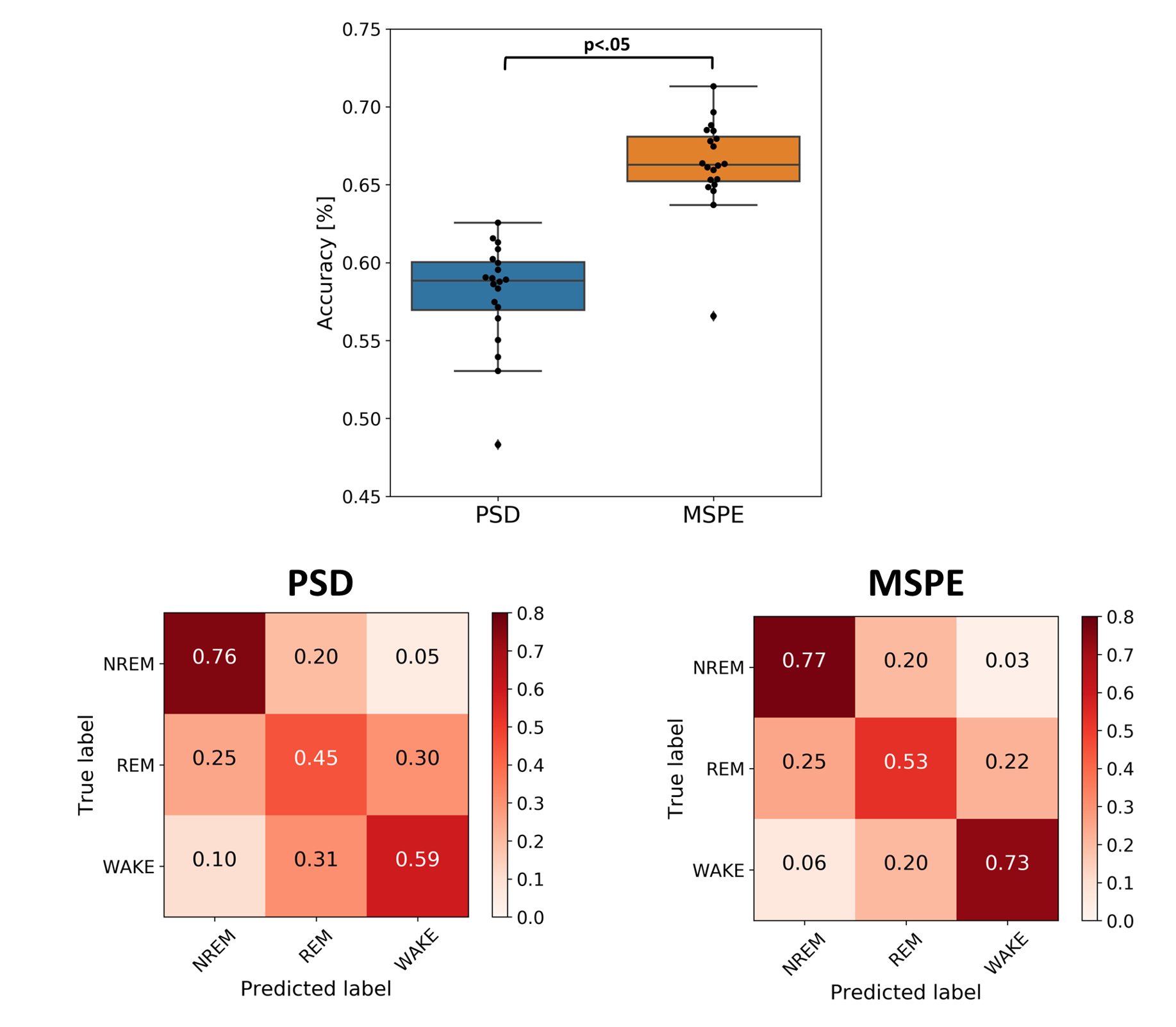

Supplement: S1 Fig — Multiscale permutation entropy as compared to PSD boosts discrimination of sleep stages. MSPE improves classification especially of WAKE, also REM and slightly NREM class (note the diagonals of the lower panels). (TIF) [file pone.0224521.s001.tif]

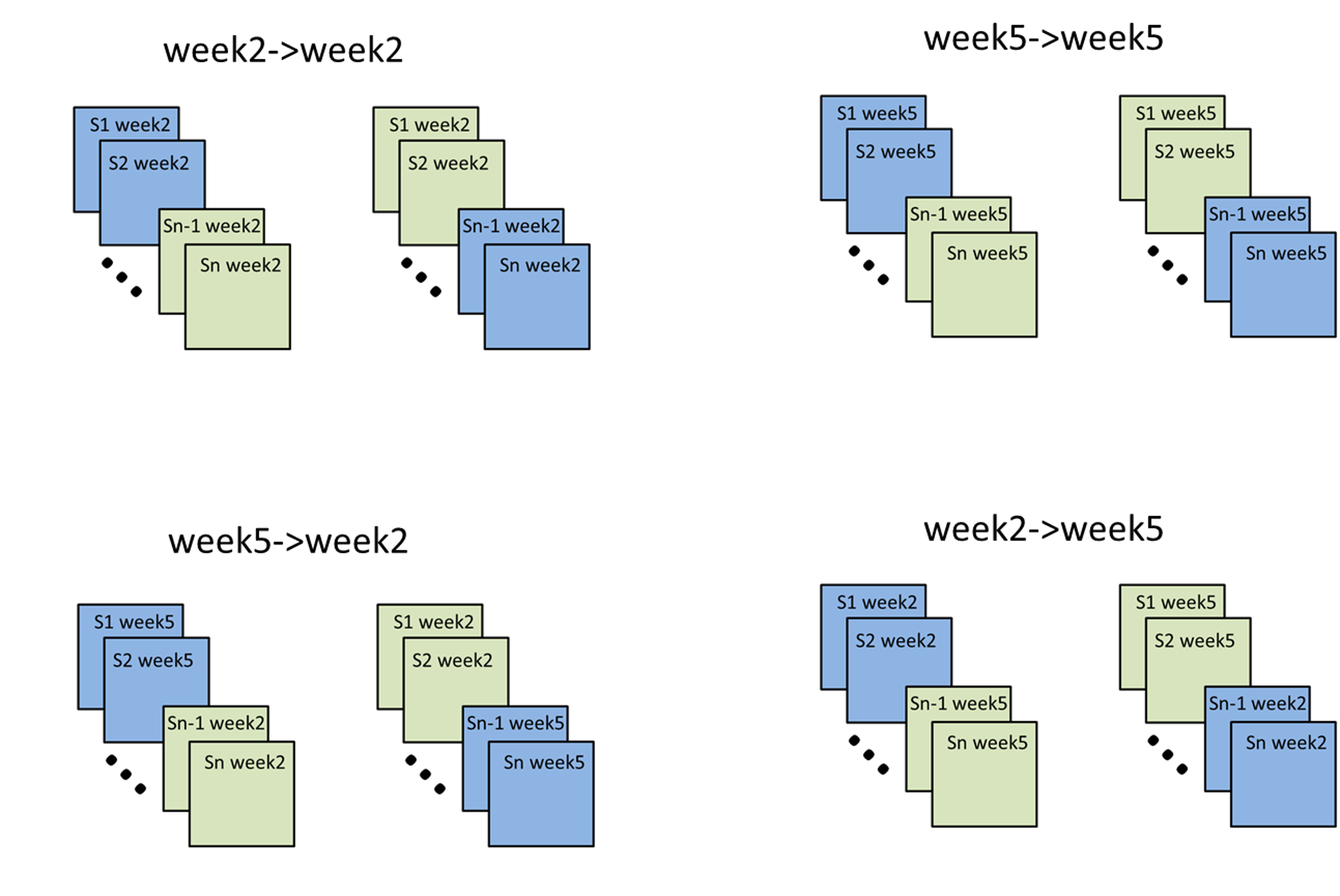

Supplement: S2 Fig — Splitting into training (in blue) and testing (in green) sets was performed within sessions (upper row) or across sessions (lower row). Note, that half of the subjects are used to train and half to test (two-fold cross validation), with both sessions of a single subject (week 2 and 5) always being in separate sets. (TIF) [file pone.0224521.s002.tif]

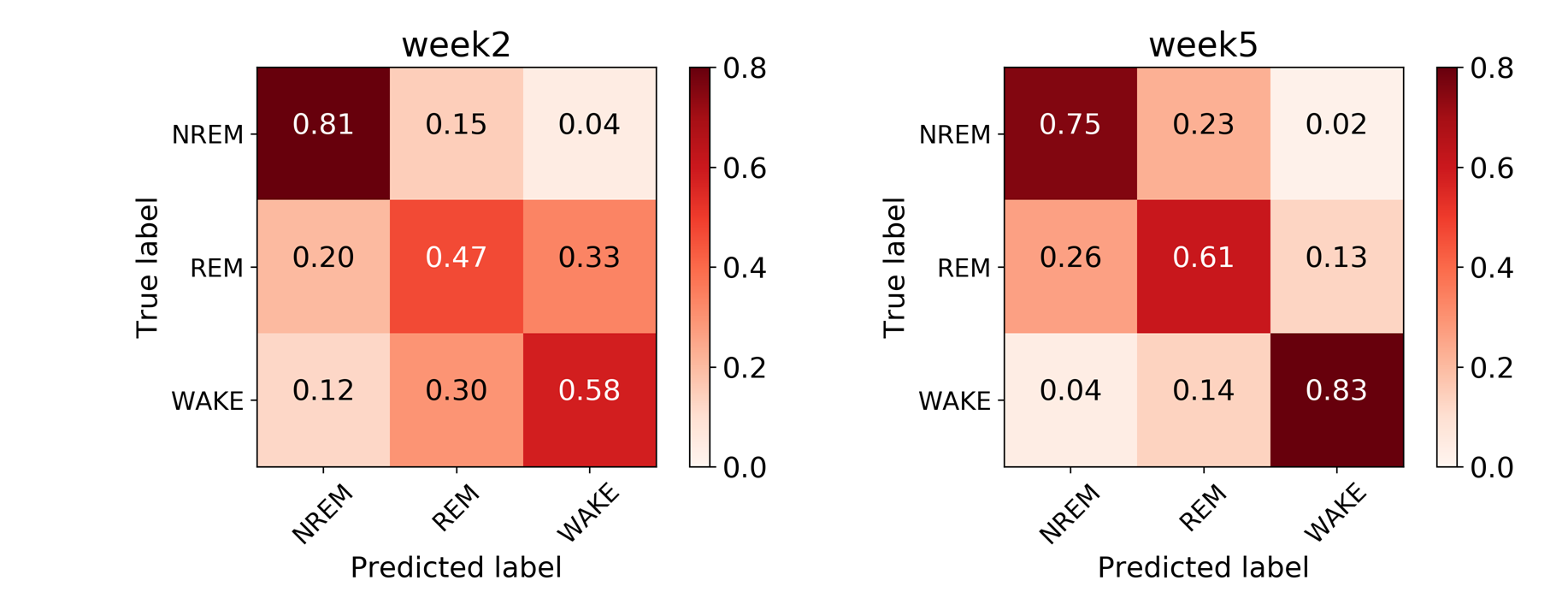

Supplement: S3 Fig — Note the off-diagonals showing limited proportion of NREM falsely classified as WAKE (on average 3%) and similarly WAKE falsely classified as NREM (on average 8%). (TIF) [file pone.0224521.s003.tif]

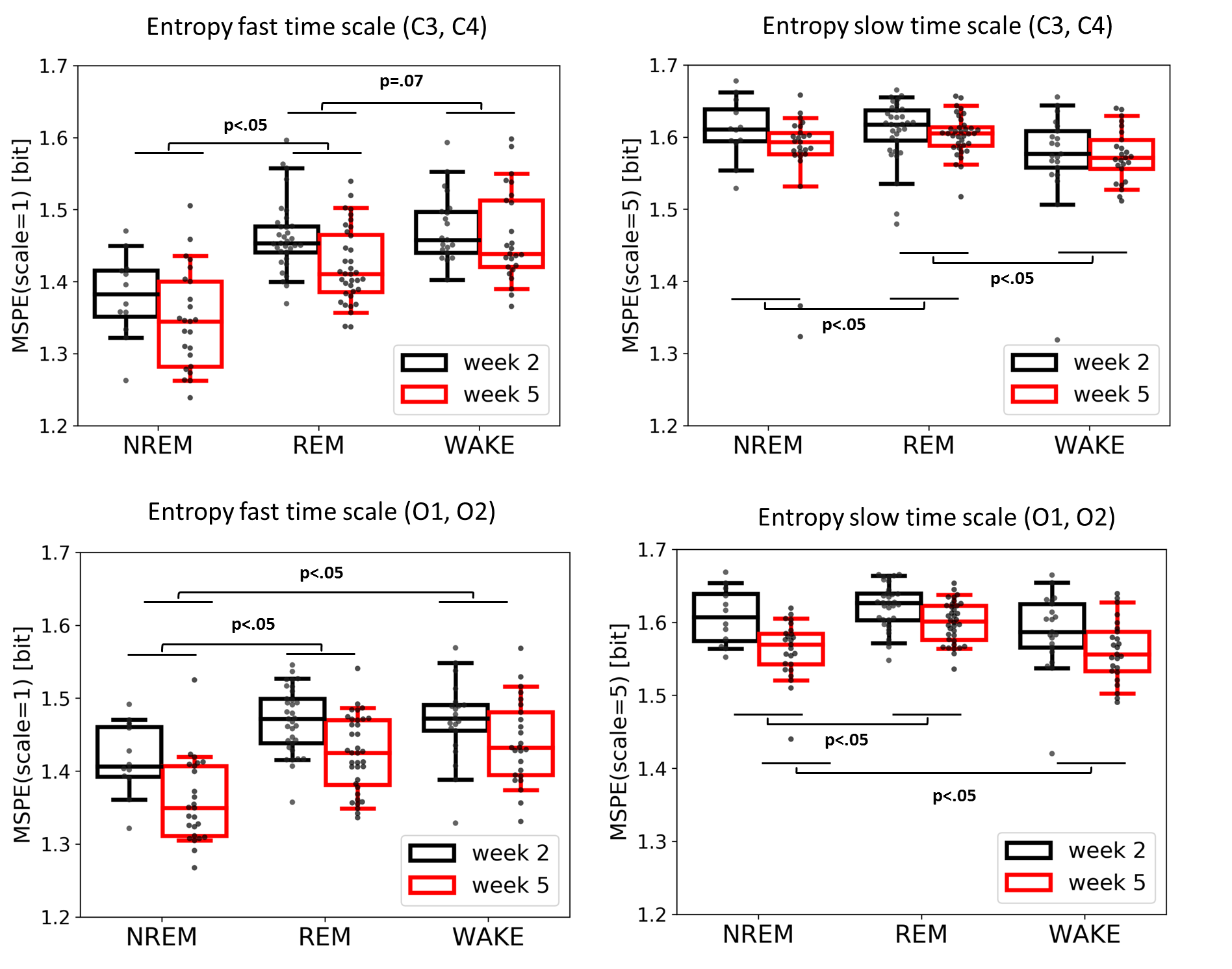

Supplement: S4 Fig — Note that overall entropy at the fast temporal scale is lower over frontal (see Fig 3, main text) as compared to both central and occipital channels (left panels). (TIF) [file pone.0224521.s004.tif]

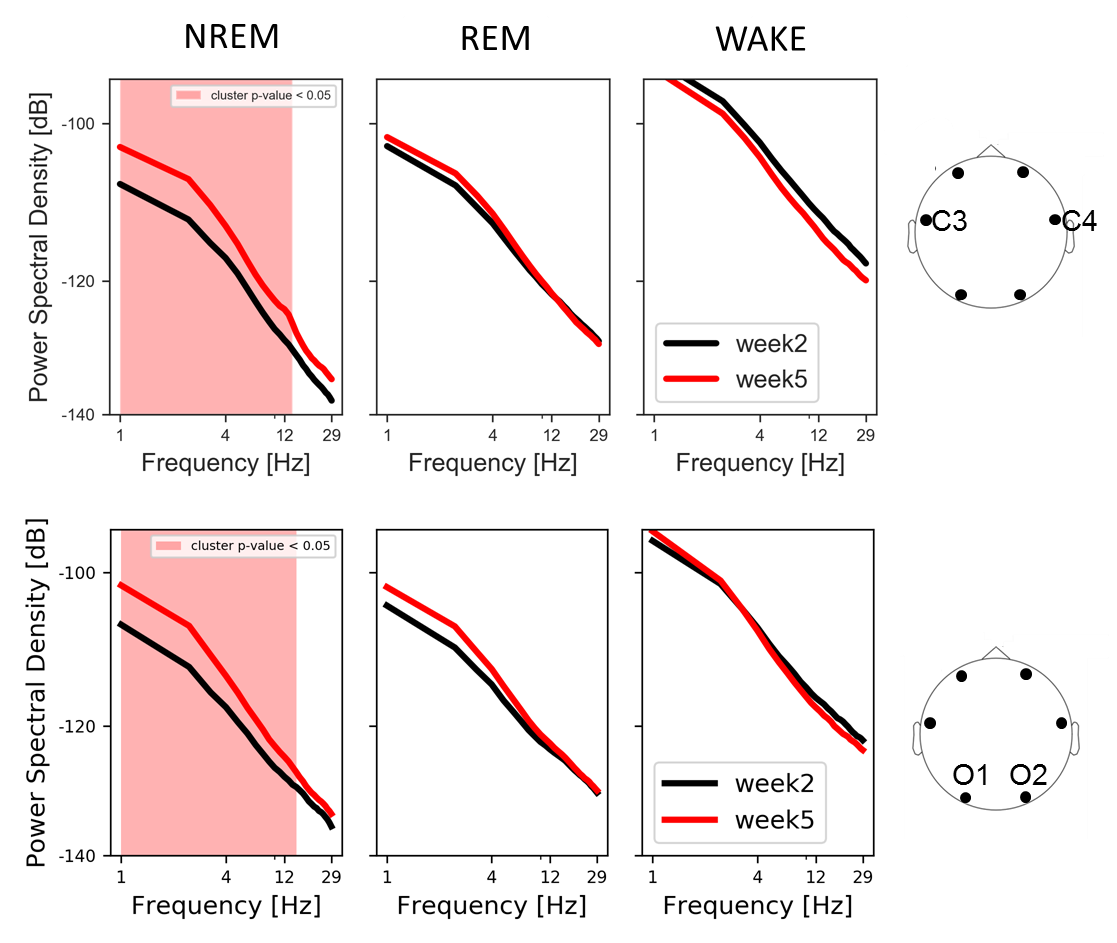

Supplement: S5 Fig — The shaded area shows statistical difference between week-2 and week-5. Note that similarly to frontal channels (main text), there is a clear difference in PSD also for central channels during NREM. (TIF) [file pone.0224521.s005.tif]

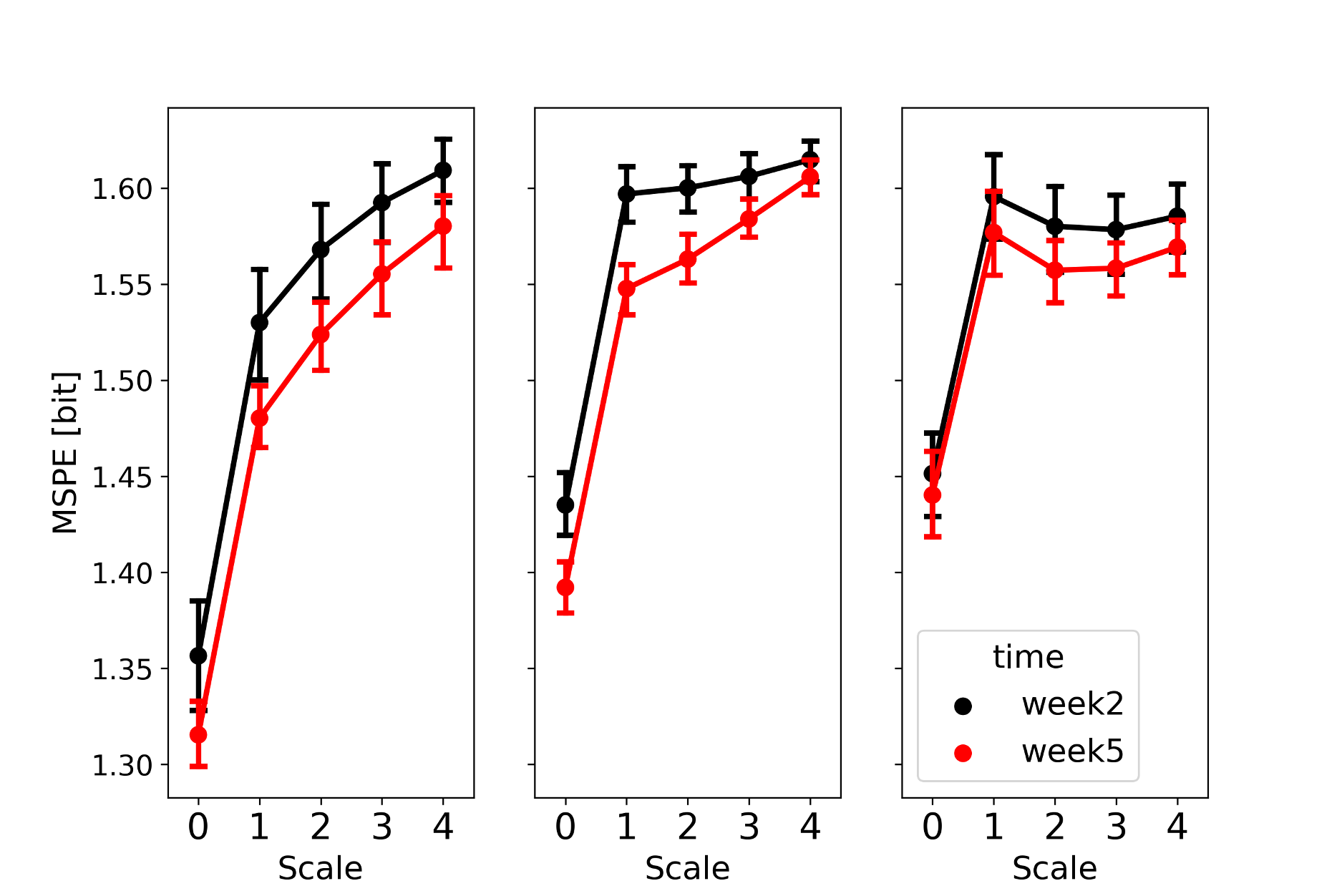

Supplement: S6 Fig — Points represent the averages and error bars show the 95% bootstrap confidence intervals. Note that results for scale = 1 and scale = 5 (X-axis) correspond to the results presented in the main text. Maximal entropy in NREM and WAKE (also REM) is attained at different temporal scale (X-axis). Also, similarly to the main text there is no statistical difference in WAKE between sessions (week-2 vs week-5) across all temporal scale. (TIF) [file pone.0224521.s006.tif]

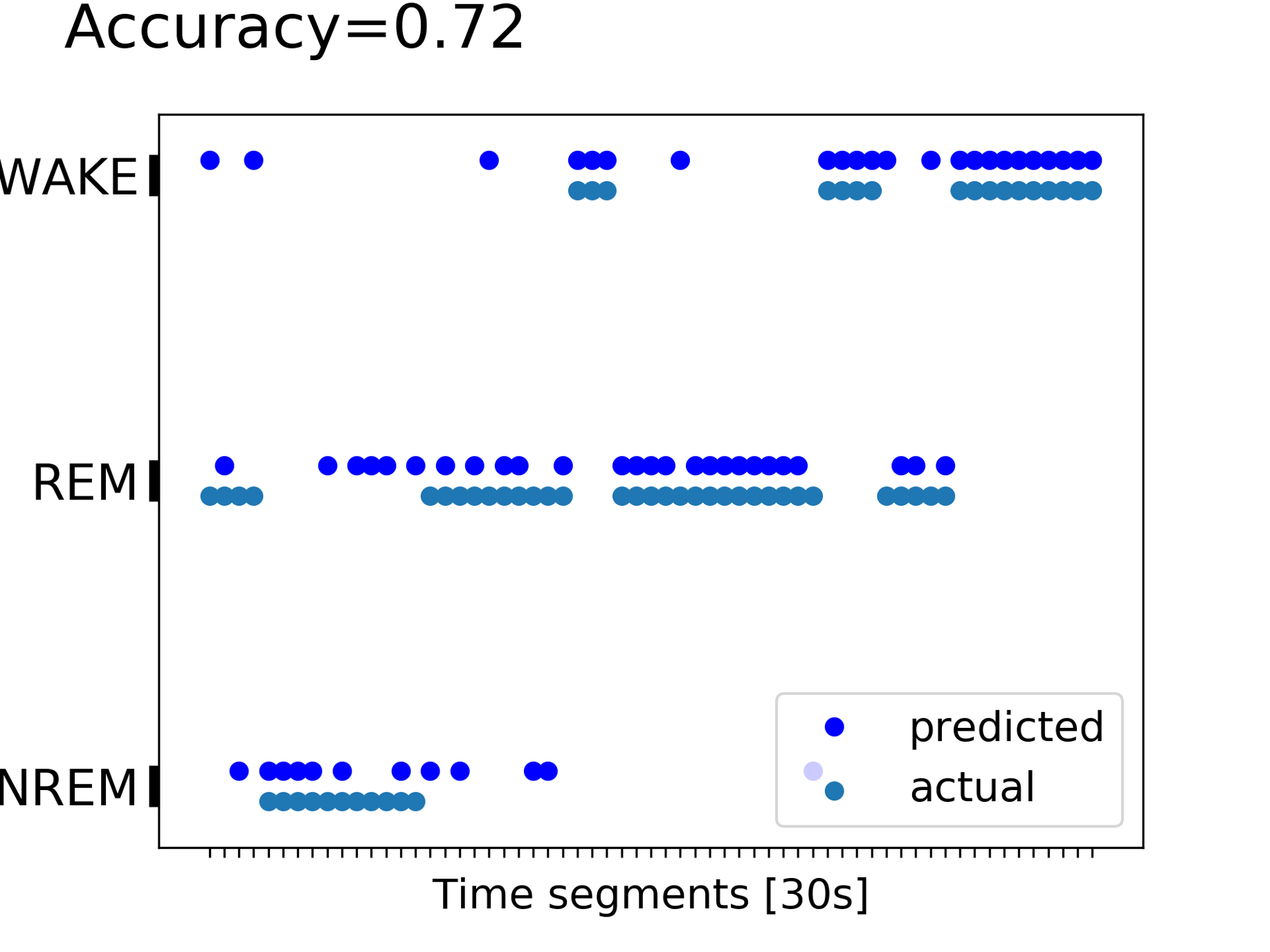

Supplement: S7 Fig — Note the limited proportion of NREM epochs being falsely classified as WAKE and similarly WAKE classified as NREM. (TIF) [file pone.0224521.s007.tif]

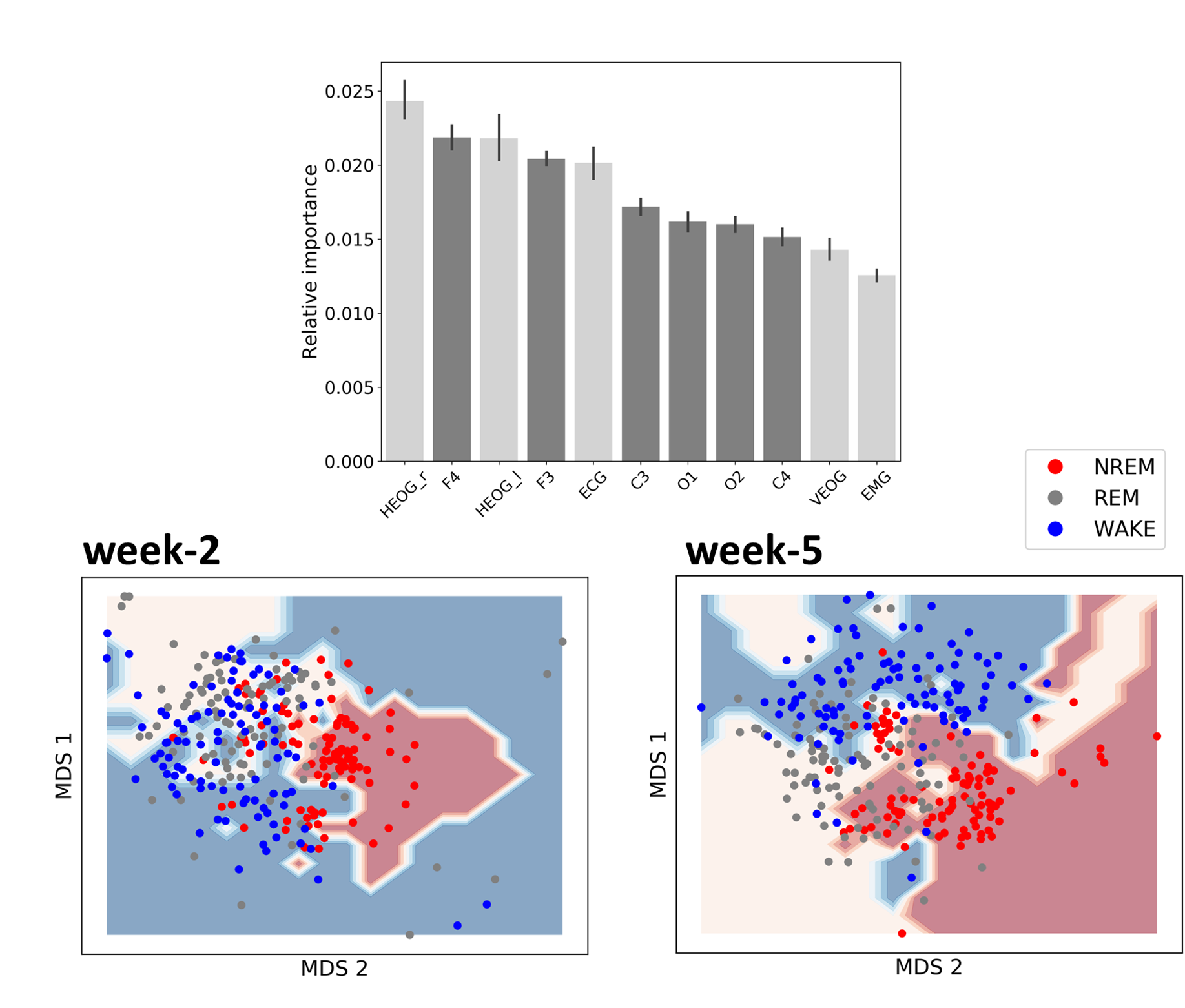

Supplement: S8 Fig — Horizontal EOGs, frontal channels as well ECG contribute most to the sleep classification (upper panel). For visualization purposes multidimensional scaling was used to reduce dimension of the MSPE data to two (lower panel, X and Y axis). Points represent epochs (N = 100 for each class), colors (red, green and blue) represent true class labels and shading (pink, light blue and light grey) shows the decision boundary. Note that in week-5 (right panel) there is more apparent overlap between the true class labels (points) and the predictions (shading) as compared to week-2, which agrees with higher classification accuracies for week-5. (TIF) [file pone.0224521.s008.tif]

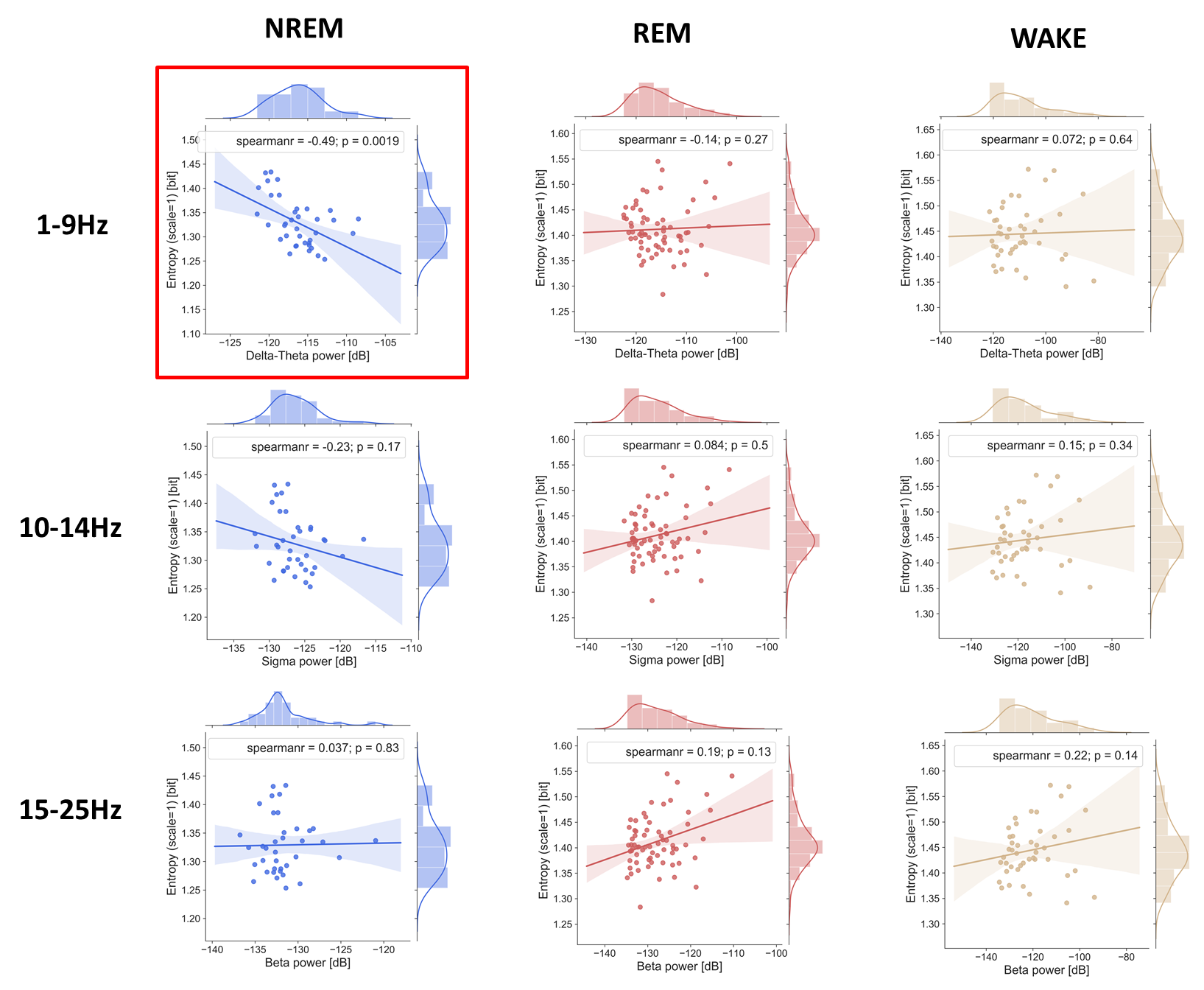

Supplement: S9 Fig — Spectral features correspond to the average power values within three frequency ranges (rows). Solid red line indicates significant results. Note negative correlation between entropy and delta-theta band power during NREM. (TIF) [file pone.0224521.s009.tif]
