# Supplementary material for: On the development of sleep states in the first weeks of life
Source: PLoS One. 2019 Oct 29;14(10):e0224521. doi: 10.1371/journal.pone.0224521 (PMC6818777; doi:10.1371/journal.pone.0224521)
Supplement: Attachment — Submitted filename: Response_to_Reviewers.docx [file pone.0224521.s011.docx]

Laboratory for Sleep, Cognition and Consciousness Research,

Centre for Cognitive Neuroscience & Department of Psychology

University of Salzburg,

Salzburg, Austria

October 9th, 2019

Dear Editor,

Once again, we thank the reviewers for their careful reading and valuable comments. We have edited the manuscript to address all the points raised.

Please find below a detailed point by point response to all comments. The reviewer’s comments are in italics, our replies start with ‘Reply’, line numbering refers to this manuscript file: Revised_Manuscript_with_Track_Changes.docx

Sincerely,

Tomasz Wielek & Manuel Schabus

On behalf of all authors

1. *A more thorough discussion of decoding sleep stages from physiological channels is still missing. While the potential influence of physiological noise on the interpretation of frontal EEG recordings has been adequately noted in multiple places, the decoding results warrant a specific discussion. I recommend noting that development of sleep patterns is not only associated with neural, but also with physiological changes (e.g., eye movement output) and that these features may be crucial markers for the manually labeled sleep stages. With the provided data, it appears wrong for readers to infer that neural differences are driving classification results and this should be made explicit.*

Reply: We agree with the reviewer and thank for the suggestion. The revised manuscript (LINE 555-558) reads:

*“*Finally yet importantly we found that horizontal EOGs, frontal brain channels as well ECG contribute the most in the classification. This suggests that development of sleep patterns is not only associated with neural, but also with physiological changes (e.g., eye movement) and that these features may be crucial markers for the manually labeled sleep stages.”

2. *“On average 5-weeks old newborns spend a higher percentage of total time (19%) in NREM sleep as compared with 2-weeks old babies (11.5%). […] A significantly larger proportion of participants showed NREM during week-5 (66%) as compared to week-2 (35%) […] Using paired-samples (by including only those subjects that actually show given sleep-state in both sessions), we found no significant effects differences in the median duration of classes from week-2 to week-5”. Were pair-wise statistics performed on the initial (overall) data? If these differences are significant, wouldn’t the inference be that cross-sectionally, NREM sleep increases, but not longitudinally within person? If so, this should be noted as much of the discussion ascribes the observed neural effects to an increase in NREM sleep and an associated increase in slow waves.*

Reply:	To compare time in a given sleep stage across sessions we used ‘standard’ pair-wise statistics which limits sample to only those subjects that actually show given sleep stage in both sessions (and in consequence reduces sensitivity/statistical power). I contrast, testing entropy changes we used more sophisticated mixed models, better preserving statistical power in the presence of missing data. We agree however that additional comment would be useful here and add following sentence in the Discussion. LINE 454-457:

*“*However, it is worth mentioning that testing longitudinally within subject, we found no	significant changes in the percentage of NREM. It was likely due to limited sensitivity in this	measure, magnified by small number of available subjects with NREM during week 2. “

3. *Thank you for adding information on when the recordings were taken. I am still a bit unclear about the exact recording setup. Were mothers asked to perform nursery rhymes as acoustic stimulation every approx. 5 min? More information on this would be appreciated. The fact that the data were not recorded during continuous night-time sleep should also be repeated in the Introduction and Discussion section to avoid misunderstandings.*

Reply: The presented auditory stimuli were pre-recorded, so mothers were not performing any task during PSD recording of their babies. The 3min or 5min periods mentioned in the text were achieved by repetitive presentation of a nursery rhyme that lasted approx. 41s each. We have modified the method section; LINE 148. Also additional clarification has been added in the Introduction (LINE123-125):

“It has to be mentioned that the reported data were not recorded during continuous night-time sleep period, thus may differ from natural sleep in newborns.”

With regard to the Discussion we believe that its first paragraph already provides sufficient clarification in this respect, thus no further changes were implemented. It reads as follows:

“The main focus of this study was the development of an automatic sleep staging technique in order to sleep stage newborn sleep as early as 2 to 5 weeks after birth. As babies at this age also sleep a significant proportion of time (and irrespective of environmental stimulation such as noise) we also had the opportunity to study sleep and associated brain dynamics in this early age group. However, please note that we do not claim that our newborn sleep data is necessarily representing natural sleep at that early age as auditory stimulation is ongoing half of the time in our study protocol.”

4. *Figure S9 is unclear to me. Why are the PSD curves repeated for each bin when they are identical? I am also not sure what the Figure is telling me about the presented bins as no statistical comparison is made here. This is complicated by the fact that value ranges are largely unreadable.*

Reply: We agree that Figure S9 presenting topo-plots and repeated psd curves is largely redundant. We decided to replace it with a new Figure S9 that shows correlations between sigma power and entropy values (please see the response to the point below).

5. *The discussion of sleep spindle development and anticorrelated entropy is much clearer now. Given this discussion, it would be interesting to know whether spectral differences between sessions were inter-individually anticorrelated with differences in entropy, i.e., whether spectral features relate to observed entropy effects.*

Reply: We thank the reviewer for this comment. As suggested we show correlations between spectral features and entropy at fast temporal scale (samples from the 2 sessions were merged). Suggested analysis for relative values (differences between sessions) was inconclusive due to small number of available subjects with NREM during week 2. LINE 761-764

6. *‘Percentile thresholding’. 95% percentile?*

Reply: Yes, that's right, we specified. LINE 158

7. ‘*individual amount’. I’d recommend ‘inter-individual differences.*

Reply: We thank for this comment. We corrected as suggested. LINE 226

8. *‘paired-samples’. Specific that those are ‘paired-samples t-tests’.*

Reply: We specified as suggested ‘Using paired-samples Wilcoxon test’. LINE 288

9. *“Note that only NREM shows differences in the PSD spectra between age groups and the developing 9–14 Hz peak exclusively observed at week-5 of age.” Differences in the alpha peak are hard to see and are more obvious at occipital channels (Figure S5). A log-log scaling in addition with error bars may aid visualization.*

Reply: We thank for this suggestion. We modified both Fig4 and S5_Fig by adding log-log scaling.

10. *“reveled” vs. ‘revealed’*

Reply: We corrected as suggested. LINE 329

11. *“We found an increase in oscillatory activity from week-2 to week-5”. I would recommend to change this to spectral power, as this does not appear to be a narrowband, i.e. oscillatory, effect. Also, the addition of error bars to Figure 4 would be beneficial.*

Reply: We corrected as suggested; LINE 343. Also we provided standard error of the mean to Figure 4.

12. *Starting the discussion with “Please note that” appears unnecessarily defensive and should be dropped.*

Reply: We thank the reviewer for this comment, we corrected as suggested. LINE 417

13. *“Given the rapid cap placement and movement of babies at this early age we would refrain from over-interpreting these results.” This is a puzzling statement to me, at least in its current phrasing. Are the authors concerned that cap positioning strongly varied across time points and babies? Then all of the other results in the manuscript would be equally questionable as the inferences assume that the selected channels are in comparable locations.*

Reply: We agree that the wording was confusing. There was no variation in cap positioning. For the reasons mentioned above, we decided not to include previous version of Figure S9 in the already lengthy supplementary materials.
